# Supplementary figures and images for: Timely initiation of postpartum contraceptive utilization in Sebata Hawas district, Ethiopia: A cross-sectional study
Source: PLOS Glob Public Health. 2023 Jan 25;3(1):e0001503. doi: 10.1371/journal.pgph.0001503 (PMC10021595; doi:10.1371/journal.pgph.0001503)

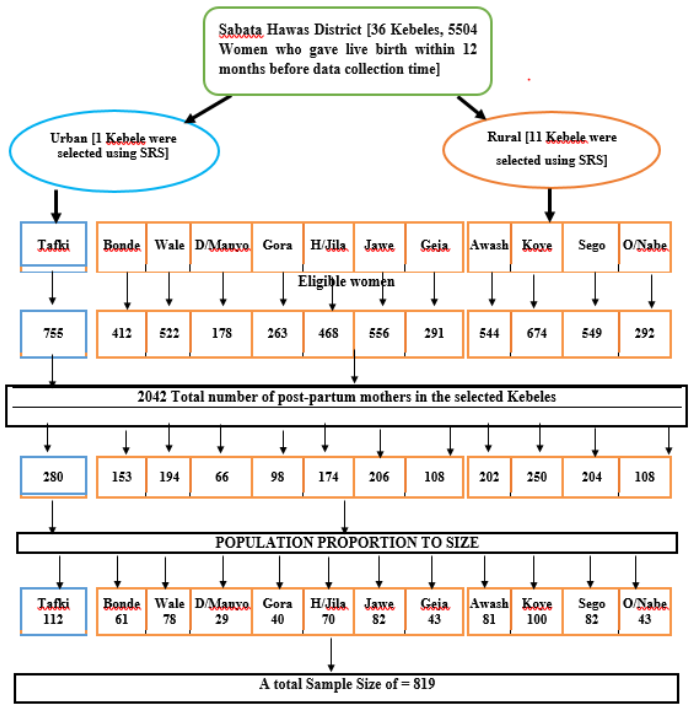

Supplement: S1 Fig — (TIF) [file pgph.0001503.s001.tif]
