# Supplementary material for: Timely initiation of postpartum contraceptive utilization in Sebata Hawas district, Ethiopia: A cross-sectional study
Source: PLOS Glob Public Health. 2023 Jan 25;3(1):e0001503. doi: 10.1371/journal.pgph.0001503 (PMC10021595; doi:10.1371/journal.pgph.0001503)
Supplement: Attachment — Submitted filename: Response to Reviewers.docx [file pgph.0001503.s002.docx]

**Response to Reviewers**

*PGPH-D-22-01272*
**Timely initiation of postpartum contraceptive utilization in Sebata Hawas District, Ethiopia: a cross-sectional study**

*Thank you for submitting your manuscript to PLOS Global Public Health. After careful consideration, we feel that it has merit but does not fully meet PLOS Global Public Health’s publication criteria as it currently stands. Therefore, we invite you to submit a revised version of the manuscript that addresses the points raised during the review process.*

**Response:** Thank you for these positive comments.

*Journal Requirements:*

1. *We have amended your Competing Interest statement to comply with journal style. We kindly ask that you double check the statement and let us know if anything is incorrect.*

**Response:** As per the editor's suggestion, we have now checked and updated based on the journal format. Which read as “**Competing interests:** The author has declared that no competing interests exist.”

2. *We are unable to open your Supporting Information file Supporting information.rar. Please kindly revise as necessary and re-upload.*

**Response:** Thank you for your advice, now we have done we done properly. “The data used in this paper are in a fully anonymised format at: <https://figshare.com/articles/dataset/S1_Data_sav/21541554> “

3. *In the online submission form, you indicated that "The data used to support the findings of this study are available from the corresponding author upon reasonable request. We have also attached as per requested." All PLOS journals now require all data underlying the findings described in their manuscript to be freely available to other researchers, either 1. In a public repository, 2. Within the manuscript itself, or 3. Uploaded as supplementary information.
   This policy applies to all data except where public deposition would breach compliance with the protocol approved by your research ethics board. If your data cannot be made publicly available for ethical or legal reasons (e.g., public availability would compromise patient privacy), please explain your reasons by return email and your exemption request will be escalated to the editor for approval. Your exemption request will be handled independently and will not hold up the peer review process, but will need to be resolved should your manuscript be accepted for publication. One of the Editorial team will then be in touch if there are any issues.*

**Response:** Again thanks, we now uploaded the data as a supplementary document.

*4. Some material included in your submission may be copyrighted. According to PLOS’s copyright policy, authors who use figures or other material (e.g., graphics, clipart, maps) from another author or copyright holder must demonstrate or obtain permission to publish this material under the Creative Commons Attribution 4.0 International (CC BY 4.0) License used by PLOS journals. Please closely review the details of PLOS’s copyright requirements here: PLOS Licenses and Copyright. If you need to request permissions from a copyright holder, you may use PLOS's Copyright Content Permission form.*

*Please respond directly to this email or email the journal office and provide any known details concerning your material's license terms and permissions required for reuse, even if you have not yet obtained copyright permissions or are unsure of your material's copyright compatibility.*

*Potential Copyright Issues:*

*Figure 1: please (a) provide a direct link to the base layer of the map (i.e., the country or region border shape) and ensure this is also included in the figure legend; and (b) provide a link to the terms of use / license information for the base layer image or shapefile. We cannot publish proprietary or copyrighted maps (e.g. Google Maps, Mapquest) and the terms of use for your map base layer must be compatible with our CC-BY 4.0 license.*

*Note: if you created the map in a software program like R or ArcGIS, please locate and indicate the source of the basemap shapefile onto which data has been plotted.*

*If your map was obtained from a copyrighted source please amend the figure so that the base map used is from an openly available source. Alternatively, please provide explicit written permission from the copyright holder granting you the right to publish the material under our CC-BY 4.0 license.*

*Please note that the following CC BY licenses are compatible with PLOS license: CC BY 4.0, CC BY 2.0 and CC BY 3.0, meanwhile such licenses as CC BY-ND 3.0 and others are not compatible due to additional restrictions.*

*If you are unsure whether you can use a map or not, please do reach out and we will be able to help you. The following websites are good examples of where you can source open access or public domain maps: 
* U.S. Geological Survey (USGS) - All maps are in the public domain. (*[*http://www.usgs.gov*](http://www.usgs.gov/)*) 
* PlaniGlobe - All maps are published under a Creative Commons license so please cite “PlaniGlobe,* [*http://www.planiglobe.com*](http://www.planiglobe.com/)*, CC BY 2.0” in the image credit after the caption. (*[*http://www.planiglobe.com/?lang=enl*](http://www.planiglobe.com/?lang=enl)*) 
* Natural Earth - All maps are public domain. (*[*http://www.naturalearthdata.com/about/terms-of-use/*](http://www.naturalearthdata.com/about/terms-of-use/)*)*

**Response:** Thanks for your comments, the map is drawn by Authors using <https://dhsprogram.com/data/dataset/Ethiopia_Interim-DHS_2019.cfm?flag=1> publicly available data. Now we have mentioned the link of the data in our map legend.

*Additional Editor Comments (if provided):*

*“Here are additional comments from the editor:*

*Complete English editing is required.”*

**Response:** The English language for the whole manuscript has been carefully checked by an expert.

*Abstract: Wouldn't it be more appropriate for the "OR" to be "AOR"?*

**Response:** Thank you for your advice, now it was revised.

*“L47, L120 Please spell out any abbreviations appearing in the text for the first time.”*

**Response:** As per suggested by editors, we have spelled out any abbreviations appearing in the text for the first time. For example: which s read as “Although Post-Partum Family Planning (PPFP) is an increasingly high priority for many countries, uptake and need for PPFP varies” (L45).

*“L72 Figure 1 does not show the study period.”*

**Response:** It was now revised as **“**Figure 1: Study area map of Sebata Hawas districts woreda, Oromia national regional state, Ethiopia in 2022. The map is drawn by Authors using <https://dhsprogram.com/data/dataset/Ethiopia_Interim-DHS_2019.cfm?flag=1>” (for detail see page 4)

*L77-L78 The sentence "All woman who…" should be rewritten.*

**Response:** As suggested we have now rewritten it as a separate sentence into inclusion and exclusion criteria

“**Eligibility criteria**

**Inclusion criteria:** Women who gave live birth and above 42 days of postpartum to 12 months have been included in the study.

**Exclusion criteria:** Women who were seriously ill and can’t able to respond to asked questionnaires. (for detail see page 4)

*L104-109 Explanations are needed on how "time menses returned" and "time of starting modern postpartum contraceptive" were measured. "Discussion with husband" on what?*

**Response:** Thanks for the comments. The time of menses returned was measured using the following two questions;

- The first question: Does your menses cycle returned after your recent birth? if yes
- Second question: The duration of the menses cycle returned after recent birth was asked _______________ (in a week).

To measure the time to start modern postpartum contraceptives, we used the following questions;

- When you have started modern contraceptive utilization after the recent birth? ______(in weeks)

Discussion with husband: when a woman freely discussed with her husband related to their desire to have children (pregnancy plan), family planning method, and methods of family planning use

*Method: Did the authors conduct a face-to-face interview using a questionnaire or conduct a self-administered questionnaire?*

**Response:** Thanks for your consideration, we collected that data based on face-to-face interviews using a structured questionnaire. It is not self-administrative, because almost half (51%; see Table 1) of the women are illiterate (unable to read and write). Therefore, we prefer a face-to-face method for data collection methods.

*L131-134、139、142、144、148、149　 The positions of the numbers are inappropriate. L147－149　Please rewrite the sentence.*

**Response:** Now it was rewritten and will be read as follows “Eight hundred four women participated in the study. The median age of the women was 28 years (Inter Quartile Range (IQR): 25-33). Of these, 82.2% were Orthodox followers and 87.2% were Oromo ethnics. Ninety-six percent of these women were married, 65.8% were housewives, 50.7% of them were illiterate, 52.7% had three to six family size, 85% were rural residents and 43.7% of the women had a monthly income <1500 Ethiopian Birr (ETB) (Table 1).” (see Page 6)

*L157　Is the parentheses of 32％ necessary?*

**Response:** Thank you for your comments. The parentheses are not necessary, now we have rewritten them accordingly.

*L158 Need explanation of "hamachisa".*

**Response:** Thank you for your notice;

**Hamachisa** is the Oromo cultural norm in which a woman who gives birth stays at the home and doesn’t go anywhere until a cultural leader permits or blessed them to do every daily activity. it varies from village to village and the duration of stay is also different, some finish at 2 months of giving birth while others two 6 months. (see page 5)

*L156-159 Incorrect sentence*

**Response:** Now it has been rewritten and readable as “This study revealed that the prevalence of timely initiation of postpartum contraceptive utilization was 38.6% (95%CI: 35.2 - 42.1). Some of the reasons for not timely initiating postpartum contraceptive utilization are; 32% wait for menses, 25% religious prohibition, 20% cultural reasons like hamachi, and lack of awareness about time to initiate. (Figure 4).” (see page 9)

*L162-164　Incorrect sentence*

**Response:** Now it was written as “In this study, 68.9% of the women have discussed with their husband about Family planning (Table 3).” (see page 10)

*L178　Incorrect parentheses*

**Response:** Thank you for in details comments, now it was revised

The odds of timely initiation of postpartum contraceptive utilization among women who had discussed with their husbands about family planning were 3 times (AOR:3.07, 95%CI: 1.61 - 5.84) more likely than those who had not discussed with their husbands about family planning (Table 4). (see page 11)

*L191　Since this is a cross-sectional study, "affect" is not an appropriate term. Better to use  "related to" instead.*

**Response:** As suggested by editors, now it was revised as **“**The study also identified factors related to timely initiation of postpartum contraceptive utilization.”

*The authors wrote the references in L204, but please write the relevant references after the first reason (L199-201) and the second reason(L201-203), respectively.*

**Response:** Thanks for the suggestion: now we have cited the proper reference in the proper position respectively. (see in details Page 13)

*L208―210　Incorrect sentences.*

**Response:** Thanks for the detailed comments, now it is revised as “These findings contradict with the previous studies [1-4]. This discrepancy may be the study setting, in our case the study setting is in a rural area (85.3%; see Table 1), in which the majority of the women have no formal education and generate less than 3000 birr per month; as a reason due to fair of the cost of service for contraceptive, they might delay” (see for detail page 13 & 14)

*L208-210 From the odds results, we understand that women with lower incomes were less likely to use contraception. However, the author's discussion seems to argue the opposite result.*

**Response:** It was addressed in the above **“***L208―210　Incorrect sentences”*

*L217-219　Incorrect sentence. Which is the subject?*

**Response:** As suggested by editors, now it was rewritten as **“**Women who received counseling during pregnancy about postpartum family planning were 3.7 times (AOR: 3.75; 95% CI:1.59 - 8.83) more likely to initiate postpartum contraceptive utilization on time than those not received counseling.” (see Page 14)

*L223-225　Incorrect sentence*

**Response:** Now it was revised as **“**A woman who has discussed with their husband or partners regarding family planning were 3 times (AOR: 3.0; 95%CI: 1.61 - 5.84) more likely to initiate postpartum contraceptive utilization timely than those who not discuss with their husbands.” (see Page 14)

*L226　 What exactly does "more information" mean in the context of a contraceptive?*

**Response:** Thank you for your comments. As we have indicated in the above majority of women have no formal education, as well in developing countries like Ethiopia women are most marginalized by their husbands. A husband who discusses freely with his wife about family planning may be important, especially for a woman. Because most households may not have a radio or TV in their house, in this way, discussing with their husbands can as a source of information for a woman.

*[Note: HTML markup is below. Please do not edit.]

Reviewers' comments:

Reviewer's Responses to Questions*

***Comments to the Author*** *1. Does this manuscript meet PLOS Global Public Health’s* [*publication criteria*](https://journals.plos.org/globalpublichealth/s/criteria-for-publication)*? Is the manuscript technically sound, and do the data support the conclusions? The manuscript must describe methodologically and ethically rigorous research with conclusions that are appropriately drawn based on the data presented.*

*Reviewer #1: Partly*

*Reviewer #2: Partly*

*2. Has the statistical analysis been performed appropriately and rigorously?*

*Reviewer #1: I don't know*

*Reviewer #2: Yes*

*3. Have the authors made all data underlying the findings in their manuscript fully available (please refer to the Data Availability Statement at the start of the manuscript PDF file)?

The* [*PLOS Data policy*](https://journals.plos.org/globalpublichealth/s/data-availability) *requires authors to make all data underlying the findings described in their manuscript fully available without restriction, with rare exception. The data should be provided as part of the manuscript or its supporting information, or deposited to a public repository. For example, in addition to summary statistics, the data points behind means, medians and variance measures should be available. If there are restrictions on publicly sharing data—e.g. participant privacy or use of data from a third party—those must be specified.*

*Reviewer #1: Yes*

*Reviewer #2: Yes*

*4. Is the manuscript presented in an intelligible fashion and written in standard English?

PLOS Global Public Health does not copyedit accepted manuscripts, so the language in submitted articles must be clear, correct, and unambiguous. Any typographical or grammatical errors should be corrected at revision, so please note any specific errors here.*

*Reviewer #1: No*

*Reviewer #2: Yes*

*5. Review Comments to the Author

Please use the space provided to explain your answers to the questions above. You may also include additional comments for the author, including concerns about dual publication, research ethics, or publication ethics. (Please upload your review as an attachment if it exceeds 20,000 characters)*

*Reviewer #1: Manuscript Number: PGPH-D-22-01272
Title: Why timely initiation of postpartum contraceptive utilization in Ethiopia is low? a cross-sectional study
The topic peaked out by the authors is vital for developing countries like Ethiopia where a major proportion of the population resides in a rural setting with limited health services, uneducated mothers, etc.
Thus, dealing with the actual status of timely initiation of postpartum contraceptive utilization and associated factors is important to make the tailored intervention.
However, the manuscript needs major revision in its content.
General
The typo of the manuscript needs reworks – line space, comma use, inconsistency in using upper and lower cases (both in paragraphs and headings/subheadings), and repetition of ideas.
Methods and Materials*

**Response***:* Thanks for your detailed comments and for showing your interest in our manuscript.

As suggested, now we have revised the whole manuscript in detail.

*1. Line 68 – 70, the sentence structure needs rephrasing (like ‘Based on… of which 31613 are reproductive…’*

**Response:** As suggested, now it was rewritten and read as “In the 2014 census, the total population in Sebata Hawas district was 143,695 people (73,284 males and 70,411 females), of this 31,613 are reproductive age groups.” (see Page 3)

*2. Under the subheading “study population description” – It is better to merge two sentences and rephrase it.*

**Response:** As suggested, now it was rephrased and read as “Women who gave live birth and were above 42 days postpartum to 12 months have been included in the study. A woman who was seriously ill and can’t able to respond to asked questionnaires was excluded from the study.” (see page 4)

*3. Sample size and sampling procedure - parts of the first paragraph is repeated in the second paragraph*

**Response:** Thanks for your advice, now it was edited.

*Results
- In general, the results part needs deep revision in terms of narration approach and grammar. To point out the specific areas some highlights are made on the mother document for the authors' perusal.*

*- Subheading ‘Maternal health Service utilization related’ looks incomplete*

## **Response:** As suggested, now it was rewritten as “Characteristics of the women related to maternal health service utilization” (for details see page 7)

*- Better to merge subheading ‘Partner communition and discussion’ to ‘Maternal health Service utilization related’ – consider rephrasing the sub-heading*

**Response:** As suggested, now it was merged with the subheading of “Characteristics of the women related to maternal health service utilization” which is read as “In this study, 83.6% of women give live birth. During pregnancy time, 16.9% of the women had two times ANC visits, 24.9% three times, and only 43% of women visited the recommended four times ANC visits. Seventy-nine percent of the women received counseling about family planning during pregnancy, 78.7% of the women delivered at health institution, 81% had postnatal care after the last delivery and only 7.3% of the women had seen their menses less than six weeks. In our sample data, 76.9% of the pregnancy was planned for recent births, and 74.5% of the women utilized contraceptives during study time. Of these, 46% use injectables and 43% of the women are using the implant method. (Figure 2). In this study, 68.9% of the women discussed with their husbands about Family planning (Table 3). (for detail see pages 7 & 8)

*Discussion
- Line 196 – 198 & 206 – 208: seems a repetition of the results.*

*- The discussion part needs a refinement*

**Response:** Thanks for the notice. However, the idea which is mentioned in *“Line 196 -198”* discusses:- the educational status of the women related to the time to initiation of postpartum contraceptive utilization. And “*Line 206-208*”, discusses:- women’s monthly income with time to initiation of postpartum contraceptive utilization. Therefore, now it was written in different forms.

*- The authors have mentioned one of the study's strengths is being a study of a large scale. Since the study covered one district only, what makes it large-scale?*

**Response:** We thank the reviewers, we have taken the comments as positive and we have compared them with the sample size of the previous study done in Ethiopia. Even though we incorporate a relatively large participant, the coverage area is limited to one district, based on this we have accepted the reviewers’ comments and now it is removed.

*- Line 234 & 235: Better to rephrase like “……between the independent and dependent variable could not be established.”*

**Response:** We thank the reviewers, we have taken the comments as positive and now it was revised as “First: the study was a cross-sectional study design, in which the causal relationship between dependent and independent variables could not be established.” (for detail see in page 14)

*Conclusion
- No need of repeating figures which were already given in the result part.*

**Response:** As suggested, we have now removed the figures from the conclusion and which is read as *“*In conclusion, the prevalence of timely initiation of postpartum contraceptive utilization in the Sebeta Hawas district was low.” (see page 14)

*Reference
- Need rework to make it up to the standard of a scientific paper*

**Response:** As suggested we have now revised it in detail.

*Questions
Educational status of the women, monthly income, counseling about family planning during pregnancy or delivery, menses returned time, and discussion about family planning with husband reported to improve timely initiation of PPFP. What is new here? Is it not a natural phenomenon? Do the authors encounter any findings indicating that these variables have a negative influence? What message the authors are going to convey from this study?*

**Response:** Thanks for your good views and suggestions. We have found that the above-stated variables as related factors for the timely initiation of postpartum contraceptive utilization. Even though this study focused only on one district, it points out that still much work will be needed to overcome this problem. The authors aimed to indicate that may be due to a lack of proper strategy or support to address the above factors on the low level of society, it results in a high fertility rate (5%; EHDS 2019) and maternal mortality at the national level or a low-income country like Ethiopia. Therefore, the authors recommend that much attention should be given to women, especially in a rural setting.

*Reviewer #2: Comments to the Author

This manuscript described “Why timely initiation of postpartum contraceptive utilization in Ethiopia is low? a cross-sectional study” which is an important issue to assess the timely initiation of PPFP utilization particularly in the study area. Despite the interesting scope of the research, the manuscript needs revision. Authors expected to explain and clarify the comments written below.*
**Response:** Thank you for the positive and detailed comments

*¬ Title: The title is appropriate, but needs to be clearly specify the study settings.*

**Response:** As suggested, now it was rewritten and can be read as “**Timely initiation of postpartum contraceptive utilization in Sebata Hawas District, Ethiopia: a cross-sectional study”**

*- What is ‘‘Timely initiation of contraceptive utilization’’ Is it Immediate PPFP? Contraceptive utilization within 6 weeks of postpartum period? – if yes why did you intended to conduct within 6 weeks than Immediate?*

**Response:**

*¬ Abstract
-- Background: Please add a clear rationale for conducting this study. There is a lot studied done on PPFP.*

**Response:** *we have now revised it as “*To the best of our knowledge, we have not found enough evidence on the associated factors of timely initiation of postpartum contraceptive utilization in the rural setting of Ethiopia. Therefore, this study aimed to assess the prevalence of timely initiation of postpartum contraceptive utilization and associated factors among women of childbearing age in, Oromia, Ethiopia. (see Page 1)

*“- Result: …..Timely initiation of postpartum utilization…rewrite as Timely initiation of postpartum contraceptive utilization*

**Response:** Thank you for your notice. We have done.

*- Conclusion and recommendation: write your recommendation based the findings of the study. Do you think 38.6% of PPFP utilization is considered as low?*

**Response:** We thank you for your comments. To date, there is no standard cut point, which classifies as low or high. However, relatively we compared it with previous studies done in Ethiopia as well as abroad. Based on this we classified our findings as low.

*¬ Introduction
1. L60-62: What makes your study differ from others? Please write clear justification*

**Response***:* Thank you for the detailed comments. We focused on a rural setting because in Ethiopia more than 85% of the population are living in a rural area. Those societies are partially marginalized because they may not get access to information, economically, as well as politically. Therefore, we aimed our setting at this society, which differs from the previous studies.

*¬ Methods
1. Put the study design*

**Response:** thank you for the advice. Now we have included and that read” This cross-sectional study design was conducted from March 30 to May 20, 2022.” (see page 3)

*2. Why you include women who gave birth in the last 12 months before data collection and exclude women with dead birth outcome and were less than 6 weeks of postpartum period?*

**Response:** Thank you for the comments. We did this to give a chance to all women who gave live birth in the past year. As well women with dead outcomes were excluded from the study because those women are heartbroken due to the loss of their infant. Therefore, they want to replace their lost infants soon. Third, women less than 6 weeks postpartum are excluded because those women have a chance to initiate postpartum contraceptive utilization even at end of 5 months. So, if we included those women, our results may be underestimated.

*3. Is 5504 women gave birth at Sabata Hawas District or at 12 selected areas per 12 months?*

**Response:** Thank you for the suggestion. Sebata Hawas district woreda is one of the high-density areas in the Oromia regional state. It thinks it is not as much surprising having annual growth of birth of 5504.

*4. Where do you conduct the pretest?*

**Response:** Thank you for your comments. The pre-test was conducted in the Akaki district near Sebata Hawas, which is in a different population.

*5. Please clearly write the operational definition of the study…Timely initiation of postpartum contraceptive utilization…..*

**Response:** As suggested, we have now included the operational definition as follows and also in the manuscript (for detail see page 5)

## **Operational Definition**

## **Operational Definition**

**Timely initiation of postpartum contraceptive utilization:** Starting of modern contraceptive utilization within 6 weeks of the postpartum period.

**Hamachisa:** is the Oromo cultural norm in which a woman who gives birth stays at the home and doesn’t go anywhere until a cultural leader permits or blessed them to do every daily activity. it varies from village to village and the duration of stay is also different, some finish at 2 months of giving birth while others two 6 months.

**Discussion with husband:** when a woman freely discussed with her husband related to their desire to have children (pregnancy plan), family planning method, and methods of family planning use

*6. How did you use …using contraceptives, reason for using family planning, type of family planning opted, time of starting modern postpartum contraceptive as independent variables?*

**Response:** As suggested. We have taken the timely initiation of postpartum contraceptive utilization as the dependent variable. As the reviewers mentioned in the above focus point, they are considered outcomes of interest, because they may lead to other consequences. Therefore, studying the cases may be important to reduce further burdens such as unplanned pregnancy, child mortality, and maternal death.

*7. L141-142: Received counseling about FP… When? A. during ANC? B. during labour and delivery? C. during PNC D. during all cascade of pregnancy (ANC, labour and delivery, and PNC)?*

**Response:** Thank you for the comments. Received counseling regarding family planning implies that the overall women who have received counseling about family planning during pregnancy.

*¬ Discussion
1. L186-190: the discussion needs logical justification*

**Response:** Thanks for in detailed comments. Now we have revised and that read as “However, this finding is slightly higher than previously done in Ethiopia (31.7%), Malawi (36%), Kenya (35.6%) and India (34.7%) [20, 23-25]. This difference might be due to improvement in health service delivery, the difference in study period as well as the socio-economic status of the study participants.” (for detail see page 13)

*2. L217: Counselling during pregnancy/delivery VS L141:- Counselling during pregnancy Vs Table2: Counselling on FP ….lacks consistency*

**Response:** Thanks for the notice. Now we have revised.

*¬ Conclusion:
- Add - Conclusion and recommendation section*

**Response:** Thanks for the suggestion. Now we have added a recommendation. It is read as “Therefore, health care providers should strengthen the integration of family planning services with maternal and child health services, provide health information about timely use of contraceptive and improve postnatal care follow-up after giving birth” (see in details page 15)

*- Needs to be more punchy and to the point/ findings.
? Table 2: Do you think number of live birth or number of having alive children determine utilization?*

**Response:** Thanks for the suggestion. As per our understanding, of course, it may be associated even though it needs further studies. Households that have few children may have an interest in more children and are less motivated to contraceptive utilization. Similarly, for live birth. This is the author's view may or may not, but it needs further studies to confirm.

*6. PLOS authors have the option to publish the peer review history of their article (*[*what does this mean?*](https://journals.plos.org/globalpublichealth/s/editorial-and-peer-review-process#loc-peer-review-history)*). If published, this will include your full peer review and any attached files.*


***Do you want your identity to be public for this peer review?*** *If you choose “no”, your identity will remain anonymous but your review may still be made public.


For information about this choice, including consent withdrawal, please see our* [*Privacy Policy*](https://www.plos.org/privacy-policy)*.*

*Reviewer #1: No*

*Reviewer #2:* ***Yes:*** *Mulualem Silesh*

References

1.	Teka, T.T., et al., *Role of antenatal and postnatal care in contraceptive use during postpartum period in western Ethiopia: a cross sectional study.* BMC research notes, 2018. **11**(1): p. 1-6.

2.	Semachew Kasa, A., M. Tarekegn, and N. Embiale, *Knowledge, attitude and practice towards family planning among reproductive age women in a resource limited settings of Northwest Ethiopia.* BMC research notes, 2018. **11**(1): p. 1-6.

3.	Abraha, T.H., A.S. Teferra, and A.A. Gelagay, *Postpartum modern contraceptive use in northern Ethiopia: prevalence and associated factors.* Epidemiology and health, 2017. **39**.

4.	Navodani, K., P. Fonseka, and C. Goonewardena, *Postpartum family planning missed opportunities across the continuum of care.* 2017.

5.	Dona, A., et al., *Timely initiation of postpartum contraceptive utilization and associated factors among women of child bearing age in Aroressa District, Southern Ethiopia: a community based cross-sectional study.* BMC public health, 2018. **18**(1): p. 1-9.
