# Supplementary material for: Timely initiation of postpartum contraceptive utilization in Sebata Hawas district, Ethiopia: A cross-sectional study
Source: PLOS Glob Public Health. 2023 Jan 25;3(1):e0001503. doi: 10.1371/journal.pgph.0001503 (PMC10021595; doi:10.1371/journal.pgph.0001503)
Supplement: Attachment — Submitted filename: Response to Reviewers.docx [file pgph.0001503.s003.docx]

**Response to Reviewers’ Comments**

PGPH-D-22-01272R1

Timely initiation of postpartum contraceptive utilization in Sebata Hawas district, Ethiopia: a cross-sectional study

*“Additional Editor Comments (if provided):*

*It is much better but does not fully answer some of the reviewers' comments. For example, the exclusion of dead women must be adequately stated in the paper, as this could cause bias in the study results.”*

**Response:** Thank you for these positive comments.

As suggested, we have now revised the exclusion criteria and readable as “A woman who died within 12 months after delivery, women with the dead birth outcome, seriously ill and can’t able to respond to asked questionnaires was excluded from the study” (for detail see page #4, Line 84-86)

*“Generally, the study design is written at the beginning of the methods. Therefore, I recommend that the following modifications be made.*

*- Change the subheading "Study area and period" to "Study design and settings".”*

**Response:** Thank you for your notice, now it was rewritten. (for detail see page #3, Line 69)

*“- Start the sentence of line 69 with the study design, such as "This cross-sectional study was conducted…" or "This is a cross-sectional study which conducted…".”*

**Response:** Thank you for your suggestion, now it was rewritten. (for detail see page #3, Line 68)

*The data collection methods should also be clearly stated in the methods section. Therefore, I suggest the authors make a subheading "Data collection" in the methods section and start with a statement such as "We collected data by face-to-face interview using a structured questionnaire." Also, I recommend moving descriptions of the contents of the variables collected by the questionnaires, translations of the questionnaire, and data collector training in the section of the "data collection" subheadings.*

**Response:** As suggested, we have now revised and readable as “

**Data collection**

We collected data through face-to-face interviews using a structured questionnaire. The contents of the variables collected by the questionnaires are maternal age, marital status, educational status of women, place of residence, monthly income, religion, ethnicity, occupational status of the mother, family size, time menses returned, fertility desire, using contraceptives, the reason for using family planning, type of family planning opted, time of starting modern postpartum contraceptive, ANC follow-up, family planning counseling, place of delivery, PNC, information, and discussion with their husband.

To assure the quality of the data 5% pre-test of the sample was conducted in the Akaki district near Sebata Hawas, which is in a different population. Training for data collectors on the contents of the questionnaire, the objective of the study, the method of data collection, and interview techniques was done. The collected data were checked for completeness, accuracy, and consistency by supervisors and the principal investigator. The questionnaire was prepared first in English and translated to the local language and was translated back into English. (for detail see page #5, Line 100-113)

*Review Comments to the Author

Please use the space provided to explain your answers to the questions above. You may also include additional comments for the author, including concerns about dual publication, research ethics, or publication ethics. (Please upload your review as an attachment if it exceeds 20,000 characters)*

*Reviewer #2: Thank you for your response. The topic is very important as input for policy makers, program managers and others stack holders particularly for study settings. However, still the manuscript needs revision and all comments are not addressed.*

**Response:** Thank you for showing your interest in our manuscript. Again, thanks for your detailed comments and for showing your interest in our manuscript.

*1. Why you exclude women with dead birth outcome? Do you think FP is not recommended to initiate early for those women who had died birth outcome?*

**Response:** Thank you for the suggestion, we have excluded a woman with dead birth outcomes, because those women were heartbroken due to the loss of their birth, as a result, they may want to become pregnant early to replace their lost birth. Of course, family planning is recommended. However, in a rural area, if a woman lost her infant and stays for a long time without having newly pregnant, she may think that discriminated against by society. This is a common problem mostly among the illiterate community. Having this, we excluded those women from our study population. (for detail see page #4, Line 84-86)

*2. Sampling procedure is not clear. Please put the schematic presentation of sampling technique*

**Response:** As suggested, now we have attached as a supplementary document, with a file name “S1 Scheme presentation of sampling procedure”

**Sebata Hawas District [36 Kebeles, 5504 Women who gave live birth within 12 months before data collection time]**

| **Tafki** |  | **Bonde** | **Wale** | **D/Manyo** | **Gora** | **H/Jila** | **Jawe** | **Geja** |  | **Awash** | **Koye** | **Sego** | **O/Nabe** |
| --- | --- | --- | --- | --- | --- | --- | --- | --- | --- | --- | --- | --- | --- |
|  |  |  |  |  |  |  |  |  |  |  |  |  |  |

**Eligible women**

| **755** |  | **412** | **522** | **178** | **263** | **468** | **556** | **291** |  | **544** | **674** | **549** | **292** |
| --- | --- | --- | --- | --- | --- | --- | --- | --- | --- | --- | --- | --- | --- |
|  |  |  |  |  |  |  |  |  |  |  |  |  |  |

**2042 Total number of post-partum mothers in the selected Kebeles**

| **280** |  | **153** | **194** | **66** | **98** | **174** | **206** | **108** |  | **202** | **250** | **204** | **108** |
| --- | --- | --- | --- | --- | --- | --- | --- | --- | --- | --- | --- | --- | --- |
|  |  |  |  |  |  |  |  |  |  |  |  |  |  |

**POPULATION PROPORTION TO SIZE**

| **Tafki**  **112** |  | **Bonde**  **61** | **Wale**  **78** | **D/Manyo**  **29** | **Gora**  **40** | **H/Jila**  **70** | **Jawe**  **82** | **Geja**  **43** |  | **Awash**  **81** | **Koye**  **100** | **Sego**  **82** | **O/Nabe**  **43** |
| --- | --- | --- | --- | --- | --- | --- | --- | --- | --- | --- | --- | --- | --- |
|  |  |  |  |  |  |  |  |  |  |  |  |  |  |

**A total Sample Size of = 819**

S1: Scheme presentation of sampling procedure

*3. Why did you intended to conduct within 6 weeks than immediate postpartum period? I think it’s better to rewrite the title as postpartum contraceptive Utilization*

**Response:** Thank you for the suggestion. We aimed to include a woman who have given a live birth and was above 42 days postpartum to 12 months rather than immediate postpartum. Because culturally and religiously in Ethiopia a woman will stay in the house separately after giving birth and be treated for at least 42 days. During this time a woman has less chance to contact their partner for sexual intercourse. Therefore, we assumed that timely initiation for postpartum contraceptive utilization after 42 days of birth. We would like to thank the reviewer’s suggestion. However, all authors decided to be taken the title as it is “*Timely initiation of postpartum contraceptive utilization in Sebata Hawas district, Ethiopia: a cross-sectional study*”

*4. How you consider contraceptives, reason for using family planning, type of family planning opted, time of starting modern postpartum contraceptive as independent variables?*

**Response:** Thank you for the suggestion, in our study timely initiation for contraceptive utilization is the dependent variable. If the woman is not timely initiated for contraceptive utilization, we aimed to determine the reason for not using family planning (waiting for menses return, religious reason, cultural reason, fear of side effects, etc), if they are family planning user to determine which type is more preferred in our setting, which helps for providing supplement based on their needs. For this reason, we aimed to include the above-mentioned indicators as independent variables.

*5. Received counseling is still lacks consistency throughout the manuscript. How you consider/categorized as woman had received counseling on FP or not?*

**Response:** Thanks for notice. Now we have revieced. We categorized counseling on family planning during pregnany as”Yes” if the woman were counselled during the service time, else “No”, if she was not. As we have mentioned the study setting of this research is rural area, in such area comprehensive service including training on family planning, pre –prepareness for delivery, feeding condition, etc. may not be given. Due to this we included this questionnaire to assess on average how many percent of women have received counselling during pregnany, and also used to determine does counselling will help to initiate timely postpartum contraceptive utilization?
